# Pre-emptive digitally guided pudendal block after posterior vaginal repair

**DOI:** 10.1007/s00192-020-04488-x

**Published:** 2020-09-02

**Authors:** Eva Uustal

**Affiliations:** Department of Obstetrics and Gynecology, and Department of Clinical and Experimental Medicine, Linköping University, 581 85 Linköping, Sweden

**Keywords:** Surgical techniques, Perineum, Prolapse, Urogynecology, Pudendal block, Posterior repair

## Abstract

**Introduction and hypothesis:**

The aim of this study was to establish if digitally guided pre-emptive pudendal block (PDB) reduces postoperative pain and facilitates recovery after posterior vaginal repair under local anesthesia and sedation.

**Methods:**

We carried out a prospective, randomized, double-blind trial in an outpatient surgery facility. Forty-one women between 18 and 70 years of age, scheduled for primary posterior vaginal reconstructive outpatient surgery, completed the study. The surgery was performed using sedation and local anesthesia with bupivacaine/adrenaline. At the end of surgery, 20 ml of either ropivacaine 7.5 mg/ml or sodium chloride (placebo) was administered as a digitally guided PDB. The primary aim was to establish if PDB with ropivacaine compared with placebo reduced the maximal pain as reported by visual analog scale (VAS) during the first 24 h after surgery. Secondary aims were to compare the duration and experience of the hospital stay, nausea, need for additional opioids, and adverse events.

**Results:**

PDB with ropivacaine after local infiltration with bupivacaine/adrenaline after outpatient posterior repair did not significantly reduce maximal postoperative pain, need for hospital admittance, nausea, or opioid use. Mild transient sensory loss occurred after ropivacaine in two women. Two women the placebo group were unable to void owing to severe postoperative pain, which was resolved by a rescue PDB.

**Conclusions:**

When bupivacaine/adrenaline is used for anesthesia in posterior vaginal repair, PDB with ropivacaine gives no benefit regarding postoperative pain, recovery or length of hospital stay. Rescue PDB can be useful for postoperative pain relief.

## Introduction

Symptoms of vaginal globus sensation, incomplete rectal emptying and a sensation of wide vaginal hiatus can occur after delivery [1, 2]. These symptoms can affect the quality of life negatively. If conservative therapy with physiotherapy and stool modification are not enough, repair of the posterior vaginal wall and perineal muscle attachments can alleviate these symptoms [3]. However, the perineum and vagina are densely innervated by the pudendal nerve and the first postoperative days can be painful. If paracetamol and nonsteroid anti-inflammatory drugs (NSAIDs) do not give sufficient pain relief, opioid treatment can be used. Although effective for pain, the negative side effects of opioids are inconvenient for outpatient vaginal surgery. Vomiting produces a strain on the pelvic floor that is especially painful after pelvic floor surgery. Nausea and dizziness make it hard for the patients to get mobilized after surgery and can necessitate overnight care.

In Sweden there is a national shortage of hospital beds. If surgical procedures can be feasibly performed on an outpatient basis, access to treatment can be maintained. In that context it is important to facilitate a smooth postoperative recovery.

Pudendal nerve block (PDB) and local anesthesia have been shown to be effective for pain relief after posterior vaginal surgery and other procedures in the area as a complement to general or spinal anesthesia [4–6]. At our outpatient surgery department, we have been providing trans-gluteal digitally guided PDB after posterior repair in sedation with bupivacaine adrenaline used for local anesthesia and hydrodissection. The digitally guided trans-gluteal route for pudendal block was introduced with posterior mesh surgery. Bupivacaine is used for hydro-dissection because it is commercially available with added adrenalin for hemostasis to minimize blood loss and facilitate surgery. Ropivacaine was chosen for post-surgery PDB because it is less likely than bupivacaine to penetrate large myelinated motor fibers and produce motor block [7]. Side effects of PDB are rare and the procedure is easy to perform (Fig. 2). The rationale behind using this additional PDB was to provide adequate pain control in the first few hours after surgery, giving the women the chance to travel home before needing opioid treatment with its possible side effects. We believed that the PDB facilitated outpatient surgery.

The primary aim of the study was to establish if PDB with ropivacaine after posterior vaginal repair is better than placebo against postoperative pain in our setting. The primary endpoint was the maximal reported pain at rest or at coughing, mean value per group, at any measuring point up to 24 h after surgery. A difference of two VAS units was stipulated to indicate a clinically significant benefit [8].

As pain is a subjective entity, we also collected data on proxy variables for pain to corroborate data on the benefit of the intervention.

The secondary aims were to establish whether there were any differences between the study groups regarding the duration and experience of the hospital stay, nausea, vomiting, need for additional opioids, occurrence of adverse events, and micturition disturbances.

Our hypothesis was that PDB is beneficial for pain relief after posterior vaginal repair under local anesthesia and sedation, by reducing maximal mean postoperative pain and thereby shortening hospital stay and reducing nausea, vomiting, adverse events, and micturition disturbances. The objective of this study was to investigate if our hypotheses were valid. If they were, the PDB method could be recommended, and outpatient surgery facilitated in other centers.

## Materials and methods

**Fig. 1:**
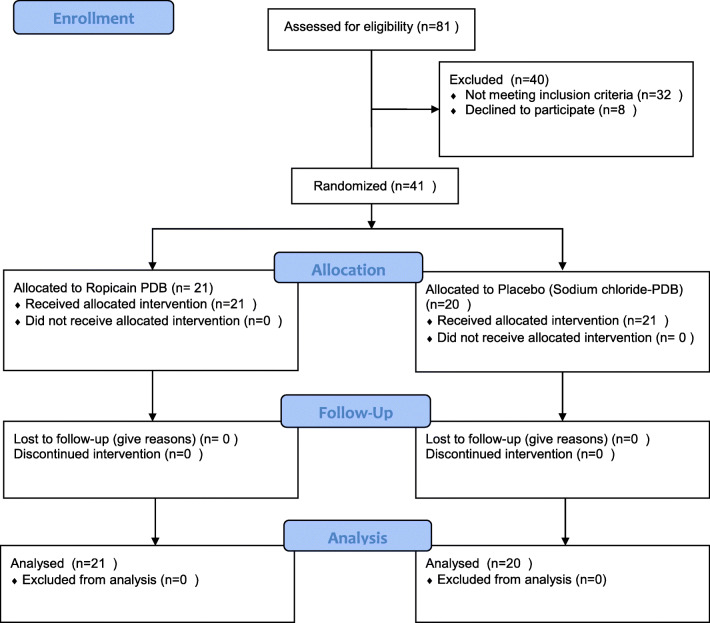
Study flow chart according to CONSORT 2010

This prospective, randomized, double-blind trial was conducted in the outpatient surgery facility at the department of obstetrics and gynecology at a university hospital during 2015–2016 (Fig. 1). It adheres to the Consolidated Standards of Reporting Trials (CONSORT) pain-specific checklist supplement criteria [9] and was monitored by Linköping Academic research center. Women between 18 and 70 years of age, scheduled for outpatient primary posterior vaginal reconstructive surgery, including perineal muscle and/or insertion point repair, were eligible for the study. Main symptoms leading to surgery are shown in Table 1. Exclusion criteria were: need for additional surgery, allergy to the study drug, pregnancy, disability affecting mobilization, concomitant heart or lung disease, mental health issues affecting study participation, insufficient language skills, chronic pelvic pain or medication for severe pain with drugs other than paracetamol and nonsteroid anti-inflammatory drugs. Inclusion was planned to continue until 40 women were included and 41 women were asked. All women were white. Demographic data are shown in Table 1.

**Table 1:** Demographic data for women receiving pudendal nerve block with ropivacaine or placebo. Values are mean, (min–max) or number. N.s = not significant

	Ropivacaine *n* = 21	Placebo *n* = 20	*t* test
Age, years	43.8 (29–68)	43.5 (24–68)	n.s.
Body mass index	24.5 (20.7–32.1)	24.3 (20.0–36.6)	n.s.
Number of smokers	1	1	n.s.
Parity	2.2 (1–4)	2.3 (1–4)	n.s.
ASA^a^ I/II	17/4	18/2	n.s.
Reason for posterior repair^b^			
Rectocele	6	9	n.s.
Digitation at defecation	8	12	n.s.
Feeling of lax vagina	14	14	n.s.

Values are mean, (min–max) or number

*n.s.* not significant

^a^American Society of Anesthesiologists physical status

^b^More than one reason could be given

After receiving written and verbal information about the study, the women who agreed to participate signed the informed consent form and were included by one of the surgeons. All patients were informed that in the case of severe postoperative pain not sufficiently alleviated by opioids, rescue PDB could be provided. Surgery was scheduled within 2 months of the screening visit. Randomization was carried out in blocks of four and individual opaque envelopes were prepared by the study monitoring agency and handled only by a research nurse not involved in patient care. As the women presented for surgery, the next-in-sequence randomization envelope was opened by the research nurse, who prepared the study drug in neutral syringes marked with the woman’s study protocol number. The trial participants, care providers, and data collectors were all blinded to allocation.

Paracetamol and ibuprofen were used for preoperative anesthesia. After voiding, walking to the operating table, being washed and dressed in sterile drapes, the patient received one dose of alfentanil and was sedated with propofol, either as patient-controlled sedation or by the attending anesthetist nurse, as preferred by the patient. The setting has been described previously [10]. Supplemental oxygen was given, and oxygen saturation and electrocardiogram were monitored. Spontaneous breathing was maintained with verbal reminders to breathe if needed. One or two doses of alfentanil could be given. Twenty milliliters of 2.5 mg/ml/5 mg/ml bupivacaine/adrenalin (Marcain ®adrenalin; Aspen Nordic, Ballerup, Denmark) was diluted with 20 ml isotonic sodium chloride (Natriumklorid; Fresenius Kabi, Uppsala, Sweden) up to 40 ml. This solution was used for hydro-dissection of the posterior vaginal wall and infiltration of the perineal area and levator muscles to provide intraoperative anesthesia and hemostasis. All dissection and deep suturing were performed with the surgeon’s finger in the anorectum. Midline plication, fascial repair and perineal muscle/and or insertion point (perineal body) repair were carried out using interrupted 3:0 polydioxanone sutures (PDS®; Ethicon, Edinburgh, UK). The vaginal wall incision was closed with continuous 2:0 polyglactin sutures (Vicryl®; Ethicon). Incisions and sutures were placed cranial to the hymenal plane. No antibiotics, intravenous fluid, vaginal packing, labial suturing or urinary catheterization were used. Perioperative data are shown in Table 2.

**Table 2:** Perioperative characteristics for women receiving pudendal nerve block with ropivacaine or placebo

	Ropivacaine *n* = 21	Placebo *n* = 20	Mann–Whitney *U* test
Operating time, minu	37 (25–55)	34 (25–60)	n.s.
Perioperative blood loss, ml	31 5–200	38 10–150	n.s.
Perioperative complications	None	None	n.s.
Total propofol, mg	302 (100–402)	230 (80–434)	<0.05
Women choosing patient-controlled sedation	13	12	n.s.
Total alfentanil administered, mg	0.39 0.25–0.50	0.41 0.25–0.50	n.s.

Values are mean, (min–max), or number

*n.s.* not significant

**Fig. 2:**
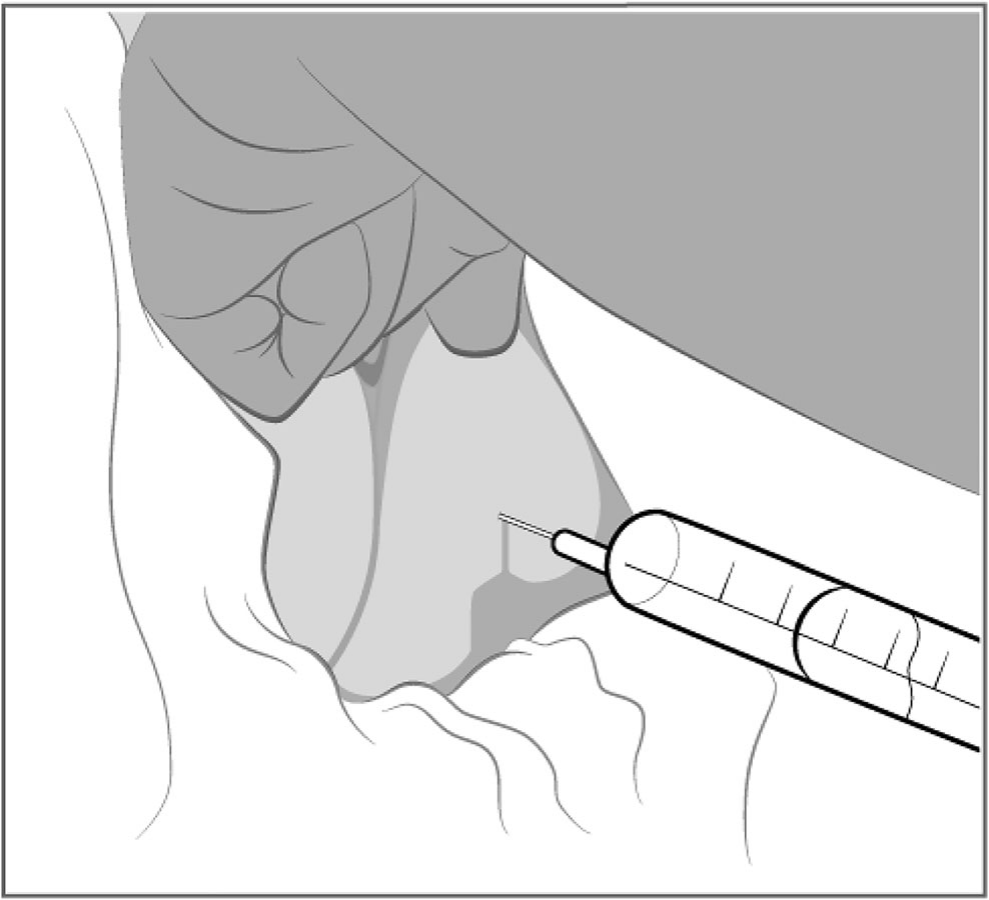
Illustration of trans-gluteal pudendal block, view of the perineum and buttocks with the surgeon’s index finger in the vagina. The needle is inserted from a point 3 cm lateral and 3 cm caudal to the anus, on the medial buttock. It is advanced through the pararectal fat toward the ischial spines while guided by a finger in the vagina. The drug is injected in a fan-shaped manner in the area around the spine after excluding intra-vasal placement by aspiration

After all sutures had been placed but before the sedation had worn off, 10 ml of the study drug, either ropivacaine 7.5 mg/ml (Fresenius Kabi, Uppsala, Sweden (study group) or isotonic sodium chloride (Natriumklorid; Fresenius Kabi, Uppsala, Sweden) (control group) was injected bilaterally for PDB. A 21 G 0.8 × 80 mm cannula (BD Medical, Stockholm, Sweden) was introduced from a point on the medial buttock 3 cm lateral and 3 cm caudal to the anus and advanced to the ischial spines (Fig. 2). The position of the tip of the needle was felt with a finger in the vagina to ensure correct injection adjacent to the pudendal nerve.

Baseline postoperative anesthesia with paracetamol and ibuprofen was given at predefined times and doses. Pain at rest and at coughing were recorded using a ten-point visual analog scale (VAS) ranging from 0 (no pain) to 10 (worst pain imaginable) before surgery, at 30-min intervals during the first 6 postoperative hours and at predefined times until 24 h after surgery.

Additional opioids were given orally or intravenously, by the postoperative care nurse, to women reporting pain of 4 or more points on the visual analog scale.

After voiding and mobilization, the women went home. Those who needed or wanted further care were admitted for an overnight stay.

The perioperative care nurse called the women 24 h after surgery to record further data and to check their general well-being. The women’s global impression of the day of surgery was collected using a VAS scale. Postoperative data are shown in Table 3.

**Table 3:** Postoperative characteristics for women receiving PDB with ropivacaine or placebo

	Ropivacaine *n* = 21	Placebo *n* = 20	Mann–Whitney *U* test
Rescue pudendal block	0	2	n.a.
Mean pain at 30 min, at cough	1.35	2.11	n.s.
Mean pain at 30 min at rest	1.15	1.74	n.s.
Mean pain at 60 min, at cough	1.50	2.21	n.s.
Mean pain at 60 min at rest	1.20	2.00	n.s.
Mean cumulative dose of opioid analgesics in the first 24 h, mg	5.8 (0–20)	5.5 (0–15)	n.s.
Unplanned overnight stay	3	2	n.a.
Time until micturition, min Hours and minutes	149 (70–255) 2 h 29 min	137 (53–250) 2 h 17 min	n.s.
Maximal postoperative pain VAS score	3.6 (0–8)	4.2 (0–10)	n.s.
Need for rescue pudendal block	0	2	n.a.
Nausea	1	0	n.a.
Vomiting	0	0	n.a.
Numbness in the legs	2	0	n.a.
Motor inhibition	0	0	n.a.
Need for intermittent bladder catheterization	2	1	n.a.
Global impression on the day of surgery, VAS (1 = good, 10 = bad)	3.5 (1.2–4.7)	3.9 (1.3–4.9)	n.s.
Time to normal activities of daily living (days)	3	3	n.a.
Patient-reported complications after 8 weeks due to anesthesia	0	0	n.a.

Values are mean, (min–max), or number

Pain presented as VAS units, 0 = no pain, 10 = maximal pain

*VAS* visual analog scale

*n.s.* not significant, *n.a.* statistical analysis not performed owing to low number

Data regarding the secondary outcomes were collected at predefined intervals during the first postoperative 24 h. Days to recovery regarding activities of daily life and patient-reported adverse events due to anesthetics were recorded after 8 weeks from the Swedish National quality register for gynecological surgery, GynOp [11].

### Statistical analysis

The hypothesis was that the intervention (PDB) would provide a two-unit reduction in mean VAS score for maximal postoperative pain in the PDB group compared with placebo and be clinically useful. With this minimum difference, a sample power of 80%, and a significance level of 0.05, the required total sample size was 40 patients. An interim analysis was performed by the monitoring agency after inclusion of 20 patients to ensure that women in the placebo group did not suffer excessive pain. This was not found to be the case and the inclusion continued. Baseline demographic data were compared using *t* test. Outcome data were compared using the Mann–Whitney *U* test. A *p* value <0.05 was considered significant. Statistical analyses were performed with SPSS for Windows, version 24 (SPSS, Chicago, IL, USA).

### Ethical approval

The study was approved by the South Eastern Sweden regional ethical review board (2014/469–31) after separate approval by the Swedish Medical Products Agency. The study was registered in Eudra-CT by registration number 2014–004236-19.

## Results

**Fig. 3:**
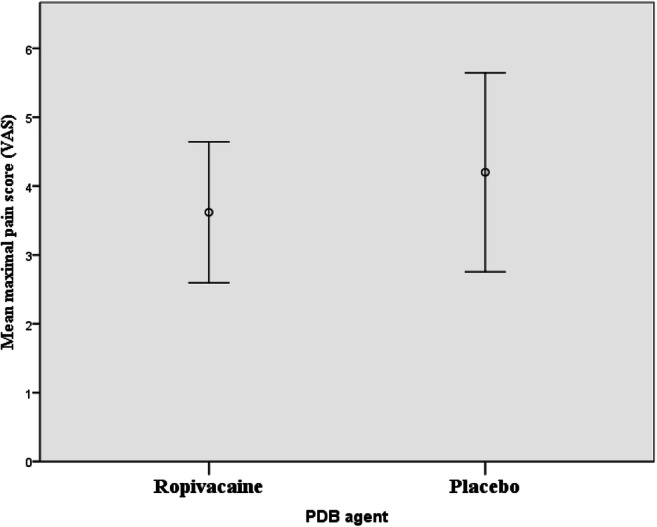
Mean maximal postoperative pain score (VAS) according to PDB agent. 95% error bars. *VAS* visual analog score, *PDB* pudendal nerve block

Addition of ropivacaine PDB to local infiltration with bupivacaine/adrenaline in posterior vaginal surgery did not significantly reduce mean postoperative maximal pain (Fig. 3). The difference of 0.6 VAS points did not reach the stipulated value of two VAS points for clinical usefulness and our hypothesis was refuted. Nor were there any differences in the secondary outcomes: duration and experience of the hospital stay, nausea, vomiting, need for additional opioids, occurrence of long-term adverse events, or micturition disturbances (Table 3).

Five out of 41 women (12%) were admitted to overnight stay because of pain or dizziness, 2 and 3 from the respective groups. The mean age of these 5 women was 34 years (range 24–44) compared with 43 years in the whole study group.

Mean doses of supplemental opioids were similar in the two groups. Two women in the placebo group were given a rescue PDB with ropivacaine owing to severe postoperative pain causing an inability to void. Pain relief and spontaneous micturition were achieved in both women after rescue PDB. Transient sensory loss of the anterior proximal thigh skin occurred after ropivacaine in 2 women. This was resolved within 6 h. No other adverse events related to the pudendal injection procedure were observed. The amount of propofol used during surgery was higher in the ropivacaine group.

The results are presented in Tables 1–3. Demographic data of the two groups were comparable.

## Discussion

In our setting, PDB after posterior vaginal repair did not significantly improve the postoperative process. This disproved our hypothesis and was surprising. Apart from the need for rescue PDB in in the placebo group and transient sensory loss in the ropivacaine group, there was no difference in either the primary or the secondary outcome.

One explanation could be that our method of placing PDB was ineffective. PDB can be placed with digital guidance or under fluoroscopic, nerve stimulator or ultrasound guidance [4, 12, 13]. These PDB techniques are valuable for caregivers who are not trained in, or comfortable with, rectal or vaginal digital palpation. It is possible that digitally guided injection is inferior to other methods that have shown effect. However, in traditional obstetric PDB placed by midwives and obstetricians, using the finger to identify the pelvic landmarks is a time-tested and feasible way of ensuring optimal placement of the local anesthetic around the pudendal nerve. Also, a recent study comparing digitally guided transvaginal PDB with ultrasound guided trans-gluteal PDP for pudendal neuralgia showed these methods to be equally as effective [14]. Our digitally guided trans-gluteal technique was effective for pain relief in the two women needing rescue PDB. This indicates that our placement of the local anesthetic may not be the problem.

A more plausible explanation for the lack of effect of additional PDB could be the long-acting effect of the bupivacaine/adrenaline used for preoperative hydro-dissection and infiltrative anesthesia. The injections given close to the pudendal nerve area may have provided patients in both study groups with lasting pain relief. In the studies showing the effect of pre-emptive PDB after gynecological surgery, only general or spinal anesthesia has been used [4, 15, 16]. In our experience [10], and supported by others [16, 17], infiltration of local anesthetics with a vasoconstricting agent in vaginal surgery is useful for dissection planes and hemostasis, in addition to the anesthetic effect. Using a long-acting anesthetic such as bupivacaine with adrenaline for infiltration seems to obviate the need for additional PDB and can be recommended.

The need for an overnight stay was around 10% in both groups. Whether PDB with ropivacaine was given or not could not be shown to make any difference. The women needing an overnight stay were younger than those returning home. This is in accordance with the literature, where younger age is associated with more postoperative pain [18, 19]. An outpatient surgery setting needs to provide the possibility of staying overnight for some women after posterior vaginal repair.

The dose of propofol used, for both patient- and nurse-controlled sedation, was higher in the ropivacaine group. There is no obvious explanation for this fact. PDB was applied at the end of the procedure; thus, the injection pain itself did not influence the dose of propofol.

The strengths of this study include the prospective, double-blinded, randomized design and that there was no loss to follow-up. It was executed with the help of a limited staff group with long experience with the procedure and patient group, providing a consistent level of care. It addresses a clinically relevant issue of postoperative pain.

The study limitations include the small sample size. We expected a large reduction of maximal postoperative pain of the PDB and chose the sample size accordingly. We did not want to expose too many women to not receiving a PDB. The staff even voiced ethical concerns about not providing PDB to all women as the study was planned. An independent interim analysis was therefore set up to ensure that it was ethically relevant to continue the study. Another limitation is that the experience of pain differs among individuals. Using a visual analog scale to record pain does not give objective data. We tried to compensate for this limitation by studying proxy variables for pain mirroring function, such as opioid use, time to micturition and time to recovery.

The overlap in effect between the perioperatively given local anesthetic and the study drug, quite possibly blurred the effect of PDB. However, as the study was set up to evaluate the addition of PDB to our standard care [9], this overlap was unavoidable.

In conclusion, when bupivacaine/adrenaline is used for hydro-dissection and anesthesia in posterior vaginal repair, pre-emptive digitally guided PDB with ropivacaine does not improve outcomes regarding postoperative pain or recovery. With our method of posterior vaginal repair, outpatient surgery is feasible in 9 out of 10 women.

The application of the study results is that we have stopped using pre-emptive PDB in standard care and reserve it for rescue postoperative pain relief. We learned that even an intuitively valuable intervention warrants scientific evaluation.
